# Analysis of Asphalt Pavement Response to Long Longitudinal Slope Considering the Influence of Temperature Fields

**DOI:** 10.3390/ma18153670

**Published:** 2025-08-05

**Authors:** Xu Li, Jie Chen, Shuxing Mao, Chaochao Liu

**Affiliations:** 1 Guangxi Xinfazhan Commnications Group Co., Ltd., Nanning 530201, China; canon240126@126.com; 2 Department of Traffic and Transportation Engineering, Changsha University of Science and Technology, Changsha 410114, China; maoshuxing0730@stu.csust.edu.cn (S.M.); lcc@csust.edu.cn (C.L.)

**Keywords:** temperature field, long longitudinal slope, finite element, mechanical response

## Abstract

With the rapid increase in traffic volume and the number of heavy-duty vehicles, the load on asphalt pavements has increased significantly. Especially on sections with long longitudinal slopes, the internal stress conditions of asphalt pavement have become even more complex. This study aims to investigate the thermal–mechanical coupling behavior of asphalt pavement structures on long longitudinal slopes under the combined influence of temperature fields and moving loads. A pavement temperature field model was developed based on the climatic conditions of Nanning (AAT: 21.8 °C; Tmax: 37 °C; Tmin: 3 °C; AAP: 1453.4 mm). In addition, a three-dimensional finite element model of asphalt pavement structures on long longitudinal slopes was established using finite element software. Variations in pavement mechanical responses were compared under different vehicle axle loads (100–200 kN), slope gradients (0–5%), braking coefficients (0–0.7), and asphalt mixture layer thicknesses (2–8 cm). The results indicate that the pavement structure exhibits a strong capacity for pressure attenuation, with the middle and lower surface layers showing more pronounced stress reduction—up to 40%—significantly greater than the 6.5% observed in the upper surface layer. As the axle load increases from 100 kN to 200 kN, the internal mechanical responses of the pavement show a linear relationship with load magnitude, with an average increase of approximately 29%. In addition, the internal shearing stress of the pavement is more sensitive to changes in slope and braking coefficient; when the slope increases from 0% to 5% and the braking coefficient increases from 0 to 0.7, the shear stress at the bottom of the upper surface layer increases by 12% and 268%, respectively. This study provides guidance for the design of asphalt pavements on long longitudinal slopes. In future designs, special attention should be given to enhancing the shear strength of the surface layer and improving the interlayer bonding performance. In particular, under conditions of steep slopes and frequent heavy vehicle traffic, the thickness and modulus of the upper surface asphalt mixture may be appropriately increased.

## 1. Introduction

The mechanical response within pavement structures serves as a key indicator of pavement performance [1,2]. With the rapid growth of traffic volume and the increasing prevalence of heavy-duty vehicles, asphalt pavements are subjected to increasingly complex stress conditions, particularly under high-temperature environments and on long longitudinal slopes [3]. Elevated temperatures significantly raise the internal temperature of asphalt layers, leading to a marked reduction in material strength and stiffness. Under these conditions, pavements become more susceptible to rutting, cracking, and other forms of distress when subjected to heavy loads, thereby compromising road functionality and traffic safety [4]. The situation is further complicated on long longitudinal slope sections, where frequent braking and slow climbing generate substantial horizontal forces on the pavement [5]. The generation of these forces causes the temperature field and load effects of the pavement structure to be more closely replicated, resulting in a cumulative effect on the mechanical response of the pavement structure [6]. Therefore, it is essential to account for the influence of temperature fields when evaluating the mechanical behavior of asphalt pavements under long longitudinal slope conditions [7,8].

Extensive research has shown that asphalt pavements on long longitudinal slopes are especially prone to rutting, which can significantly compromise both driving safety and comfort [9,10,11,12]. Numerous studies have demonstrated that temperature-related environmental factors exert a strong influence on rutting, highlighting the need to account for internal temperature distributions within asphalt pavement structures [13,14,15,16]. Liu G et al. investigated rutting resistance across various material types and aggregate gradations [17,18,19]. Liao G et al. reported that shear stress within the asphalt layer is a key contributor to rutting, and emphasized the importance of enhancing the shear resistance of asphalt mixtures [20,21,22]. Zhou et al. found that rutting on long longitudinal slopes tends to occur in the intermediate and lower pavement layers, and that incorporating an asphalt-treated base (ATB) can effectively reduce rut depth [23,24,25].

In addition, many researchers have employed numerical simulation to examine the mechanical behavior of pavements on long longitudinal slopes [26,27,28]. Shi T et al. developed a three-dimensional finite element model using 3DMove Analysis, revealing that vehicle acceleration or braking can greatly amplify peak shear stress within the pavement [29]. These peak stresses typically occur 0–4 cm beneath the pavement surface, underscoring the critical importance of improving rutting resistance in the upper asphalt layer. Li Y et al. also constructed a three-dimensional finite element model to analyze strain responses under varying slope conditions and found that a 7% slope increased the peak shear stress by approximately 14% compared to level terrain [30]. Jun Fu et al. further examined how pavement responses vary with vehicle speed and loading, and concluded that maximum shear stress and vertical strain are directly proportional to both slope gradient and traffic load, but inversely proportional to vehicle speed [31,32].

Overall, previous studies have laid the groundwork for analyzing the thermal–mechanical behavior of asphalt pavements on long longitudinal slopes, but notable gaps remain. Many prior numerical studies simplified the loading as static, neglecting the dynamic impact of moving vehicles and therefore not capturing realistic stress conditions. In addition, the unique temperature distribution characteristics of long slope sections were often overlooked, with insufficient exploration of how temperature and load effects interact. This study simulates the in situ temperature field of a mountainous long longitudinal slopes using meteorological data from Nanning, China, and incorporates moving vehicle loads to establish a thermal–mechanical coupling model for asphalt pavement on long longitudinal slopes. Unlike previous studies, this work integrates factors such as loads, slope gradients, braking coefficients, and pavement thickness into a comprehensive analysis framework, performing a parametric analysis of how these variables influence the mechanical responses of the pavement structure under the thermal–mechanical coupling model. The results reveal the characteristics of the internal temperature distribution within the pavement on long longitudinal slopes, as well as the patterns of the pavement’s mechanical responses, thereby providing critical data to further guide the design, maintenance, and management of asphalt pavements on long longitudinal slopes.

## 2. Experimental Methods

To investigate the internal thermo-mechanical response of asphalt pavements under the combined effects of temperature fields and moving loads, a three-dimensional finite element model incorporating temperature effects and moving vehicle loads was developed using ABAQUS 2022 software. First, based on meteorological data from Nanning, China, a pavement temperature field model was established, accounting for boundary conditions such as solar radiation, atmospheric convection, and effective radiation, to reflect the internal temperature distribution of the pavement structure. On this basis, a three-dimensional asphalt pavement model on a longitudinal slope was constructed to simulate moving vehicle loads, in which dynamic tire–pavement interaction was represented by a time-dependent pressure function to realistically reproduce the contact stress evolution during vehicle movement. Furthermore, a series of parametric analyses were conducted to evaluate the mechanical responses of the pavement structure under varying conditions, including axle loads, slope gradients, braking coefficients, and structural layer thicknesses. These parameters were selected to systematically assess their respective influences on the thermo-mechanical coupling behavior of asphalt pavements on long downhill sections. Overall, this modeling framework is well-suited to achieving the research objectives, namely, evaluating the coupled pavement responses under specific operational and environmental conditions and identifying key influencing factors.

### 2.1. Three-Dimensional Finite Element Model of Pavement Temperature Field

**Figure 1:**
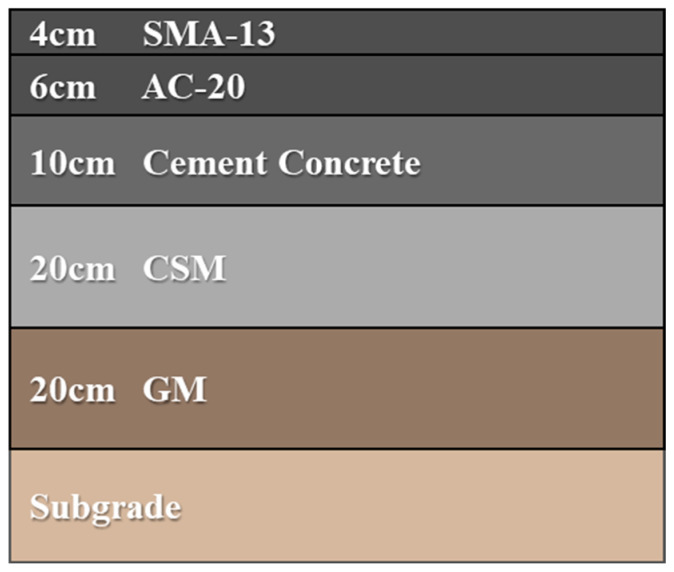
Schematic diagram of pavement structure.

This study analyzes a composite pavement structure comprising a 4 cm SMA-13 layer, a 6 cm AC-20 layer, a 10 cm cement concrete layer, a 20 cm cement-stabilized base, a 20 cm graded crushed stone layer, and the subgrade [33,34]. The pavement structure is shown in Figure 1. The model dimensions were set to 5 m × 10 m × 5 m, with the positive *X*-axis aligned with the pavement’s traffic direction, the *Y*-axis perpendicular to it, and the *Z*-axis representing pavement depth. The mechanical and thermal properties of the materials are presented in Table 1 and Table 2, respectively.

The model employs three-dimensional eight-node linear reduced integration elements (C3D8R) for meshing. The finite element mesh was constructed based on the geometric features and material distribution of the pavement structure. To balance computational accuracy and efficiency, an appropriate meshing strategy was adopted. Finer meshes were applied in critical areas—such as regions with stress concentration or high stress gradients—to capture more accurate stress and deformation responses. In contrast, coarser meshes were used in relatively uniform regions to reduce the computational cost. The effects of solar radiation, air temperature, convective heat exchange, and pavement effective radiation were described using time-dependent periodic boundary conditions. Solar radiation was modeled using Equations (1)–(3), air temperature and convective heat exchange were represented by Equation (4), and pavement effective radiation was described by Equation (5). The above boundary conditions were implemented using the user subroutines FILM and DFLUX in ABAQUS and solved through heat transfer analysis. The following assumptions were made in the process:

1. Each structural layer is assumed to be a homogeneous and isotropic solid;
2. The materials of each composite pavement layer are assumed to be tightly bonded, ensuring continuity of temperature and heat flux across interfaces;
3. Lateral temperature variation in the pavement is neglected, assuming one-dimensional heat transfer vertically downward along the traffic direction;
4. Except for specified thermal physical parameters that vary with temperature, all other thermal properties are assumed constant and independent of temperature changes.

(1) $$q(t)=\frac{a_{0}}{2}+\sum_{k=1}^{\infty }\;a_{k}cos\frac{k\pi (t-12)}{12}$$

(2) $$a_{0}=\frac{2q_{0}}{m\pi }$$

(3) $$a_{k}=\left\{\begin{array}{cc} \frac{q_{0}}{\pi }[\frac{1}{m+k}sin\left(m+k\right)\frac{\pi }{2m}+\frac{\pi }{2m}], & k=m\\ \frac{q_{0}}{\pi }[\frac{1}{m+k}sin\left(m+k\right)\frac{\pi }{2m}+\frac{1}{m-k}sin\left(m+k\right)\frac{\pi }{2m}+\frac{\pi }{2m}], & k\neq m \end{array}\right.$$

where k is the calculation order, $q_{0}$ is the midday peak radiation (W/m^2^), and m is the correction factor for solar radiation, defined as the ratio of effective sunshine duration to 12 h.

(4) $$T_{a}=\bar{T_{a}}+T_{m}[0.96\;sin\omega (t-t_{0})+0.14\;sin2\omega (t-t_{0})]$$

where $\bar{T_{a}}$ is the daily average temperature (°C), $T_{m}$ is the daily temperature variation (°C), ω is the angular frequency (rad), and $t_{0}$ is the initial phase (h).

(5) $$q_{F}=\varepsilon \sigma [{T_{1}{|}_{z=0}}^{4}-{T_{a}}^{4}]$$

where $q_{F}$ is the effective radiation of earth’s surface (W/(m^2^·°C), ε is the road surface emissivity, σ is the Stefan–Boltzmann Constant (W/m^2^·k^4^), $T_{1}{|}_{z=0}$ is the pavement surface temperature (K), and $T_{a}$ is the atmospheric temperature (K).

Nanning is located in the mountainous region of southwestern China and has a subtropical monsoon climate. The average annual temperature is 21.8 °C, with a maximum temperature of 37 °C and a minimum temperature of 3 °C. The average annual rainfall is 1453.4 mm, the average altitude is 76.5 m, and the average annual sunshine duration is 1480.4 h. Representative daily temperature data for summer and winter were selected from the annual records of Nanning City to serve as input parameters for establishing the temperature field. The summer data are presented in Table 3, while the winter data are shown in Table 4.

### 2.2. Three-Dimensional Finite Element Model of Pavement with Moving Loads

Under actual service conditions, vehicle loads on pavement structures are typically moving loads exhibiting significant dynamic characteristics. On a long longitudinal slope, vehicle acceleration and braking generate additional horizontal forces, further complicating the stress state of asphalt pavement structures. However, existing pavement design theories and analysis methods largely rely on static load assumptions and do not adequately account for the moving nature of vehicle loads and associated horizontal forces. This leads to potential discrepancies between predicted mechanical responses and actual service conditions. To more accurately simulate the effects of moving vehicle loads on pavement structures, this study developed a three-dimensional finite element model of moving loads, employing the same structural design and mesh size as the temperature field model [35].

**Figure 2:**
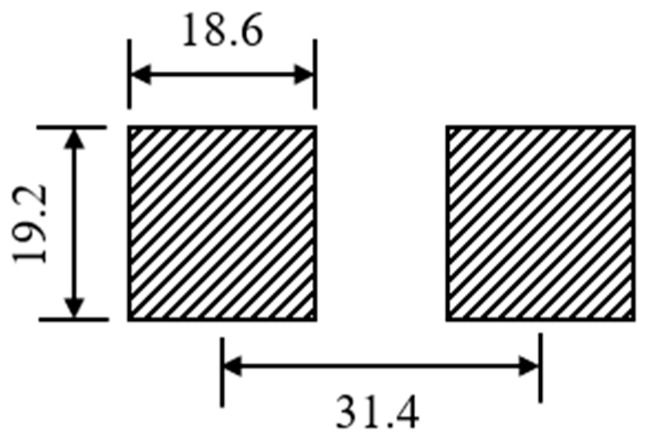
Simplified diagram of wheel load.

In pavement structure design, the tire contact area is approximated as a rectangle, simplifying a uniaxial double wheel load into two rectangular loads. Each rectangle measures 19.2 cm × 18.6 cm, with a wheel spacing of 31.4 cm, as illustrated in Figure 2. The assumed boundary conditions are as follows: the bottom surface of the model is fully constrained; the lateral faces are restricted from transverse displacement; the front and rear faces are restricted from longitudinal displacement; and full continuity is assumed at all interlayer interfaces.

**Figure 3:**
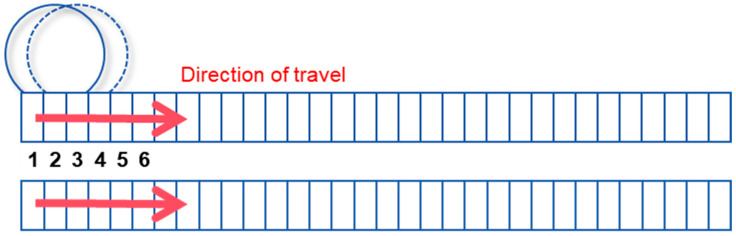
Schematic diagram of moving load.

To simulate load movement, the VDLOAD subroutine in ABAQUS was employed. A moving load band was defined on the pavement surface, with its width matching the transverse dimension of the applied uniform load and its length along the longitudinal direction equal to the load travel distance. The load band was subdivided into multiple small rectangles, each 6.4 cm long, as illustrated in Figure 3. The load gradually moves forward along the moving band through sequential analysis steps. At the end of each step, the load shifts forward by the length of one small rectangle. For example, the load initially covers rectangles 1, 2, and 3; after the first step, it covers rectangles 2, 3, and 4; it then covers rectangles 3, 4, and 5; and once the end is reached, the sequence restarts. This process repeats to simulate the moving load.

When the vehicle travels at a constant speed, the load is assumed to be a uniformly distributed vertical load. During braking, the load is considered as a combination of a uniformly distributed vertical load and a horizontal load, with the latter calculated using the following formula.

(6) T=φP

where T is the horizontal load during braking (kN), φ is the braking force coefficient, and P is the vertical load (kN).

## 3. Pavement Structure Temperature Field Analysis

### 3.1. Temperature Distribution Characteristics of Pavement Under Typical Seasonal Conditions

**Figure 4:**
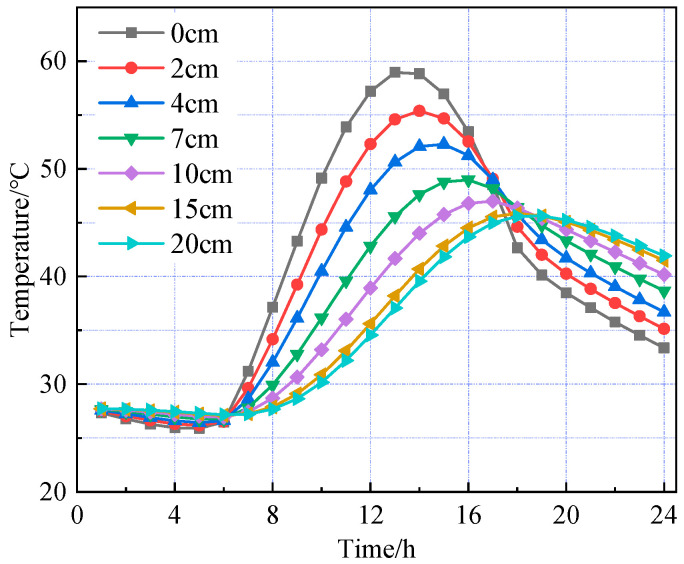
Temperature distribution at various depths during the summer season.

**Figure 5:**
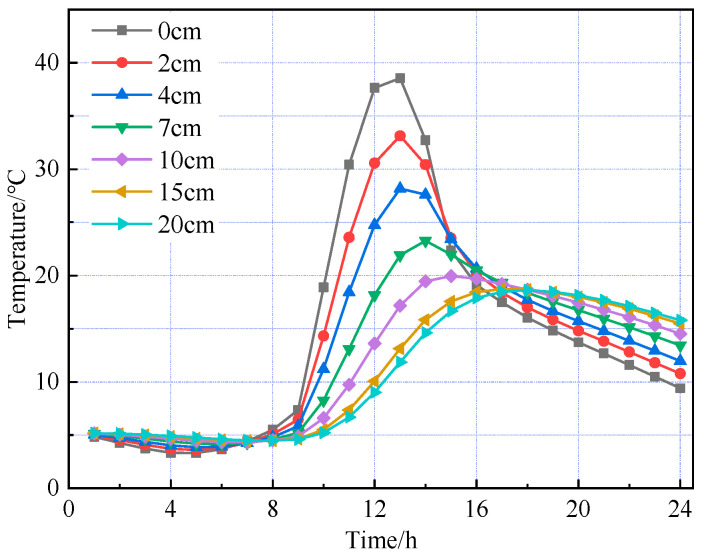
Temperature distribution at various depths during the winter season.

Based on the road surface temperature field model established above, the road structure models under summer and winter conditions were analyzed separately. The temperature changes over time at different depths within the road surface are shown in Figure 4 and Figure 5, respectively.

Figure 4 and Figure 5 show that the temperature variation trends with depth across the pavement layers are generally consistent between the high- and low-temperature seasons. As the pavement depth increases, the amplitude of temperature variation gradually decreases, with fluctuations in the asphalt concrete layer significantly greater than those in the cement concrete layer. The pavement surface is directly influenced by combined solar radiation and air temperature, absorbing part of the solar radiation and starting to warm. In the finite element simulation, it is assumed that heat transfer occurs only via one-dimensional transient conduction along the depth of the pavement structure. Therefore, as the surface temperature rises, heat flow begins to conduct downward with a delay, resulting in different pavement depths reaching their peak temperatures at varying times. During the high-temperature season, the pavement surface reaches its peak temperature of 58.95 °C at 1:00 p.m., while the bottom of the asphalt concrete layer peaks at 46.99 °C at 5:00 p.m., indicating a temperature lag of approximately four hours. From midnight to 6:00 a.m., when solar radiation is absent, temperature changes in the pavement layers are minimal. Between 6:00 a.m. and 1:00 p.m., solar radiation intensifies and ambient temperature rises, causing temperatures in all layers to increase, with the pavement surface experiencing the greatest temperature rise and fastest warming rate. From 1:00 p.m. to midnight, solar radiation gradually decreases and ambient temperature drops; however, temperature changes across the layers are uneven—the surface temperature steadily declines, the cement concrete layer first warms then cools, and temperature increases with depth within the pavement.

**Figure 6:**
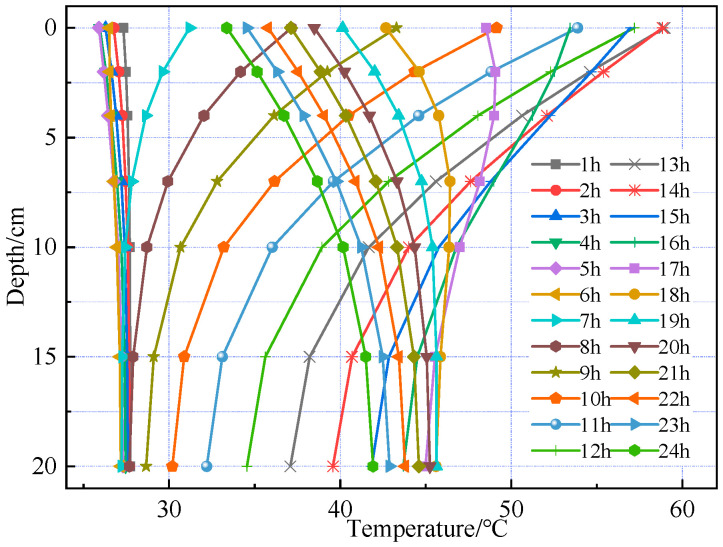
Temperature distribution at different times during the summer season.

**Figure 7:**
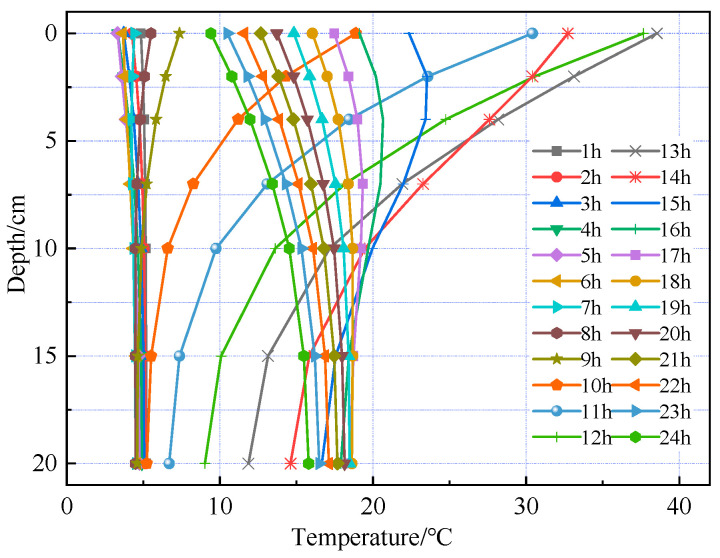
Temperature distribution at different times during the winter season.

Figure 6 and Figure 7 show that temperature variation trends along the pavement depth are generally consistent in both summer and winter. From midnight to 6:00 a.m. in summer, temperature variation with depth is minimal. Starting at 7:00 a.m., temperature variation with depth gradually intensifies, peaking at 1:00 p.m. with a maximum temperature difference of approximately 21.9 °C. The period of minimal temperature variation in winter lasts longer than in summer, extending until 9:00 a.m. Additionally, the maximum temperature difference in winter is approximately 28.6 °C, exceeding that of summer.

### 3.2. Temperature Analysis Under Different Asphalt Layer Thickness Conditions

The above analysis indicates that the asphalt mixture layer is highly sensitive to temperature changes within the pavement. Therefore, further simulations of the temperature field with varying asphalt layer thicknesses were conducted to explore how structural design parameters affect pavement temperature response and to reveal the impact of asphalt layer thickness variations on the temperature field.

**Figure 8:**
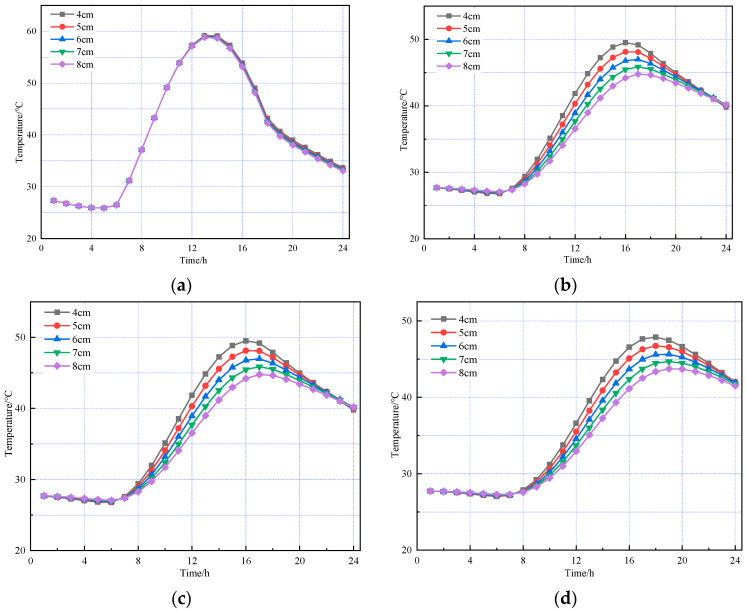
Temperature changes in pavement structures with different AC-20 thicknesses under summer temperatures. (**a**) The top of the SMA-13 layer. (**b**) The top of the AC-20 layer. (**c**) The top of the cement concrete layer. (**d**) The bottom of the cement concrete layer.

**Figure 9:**
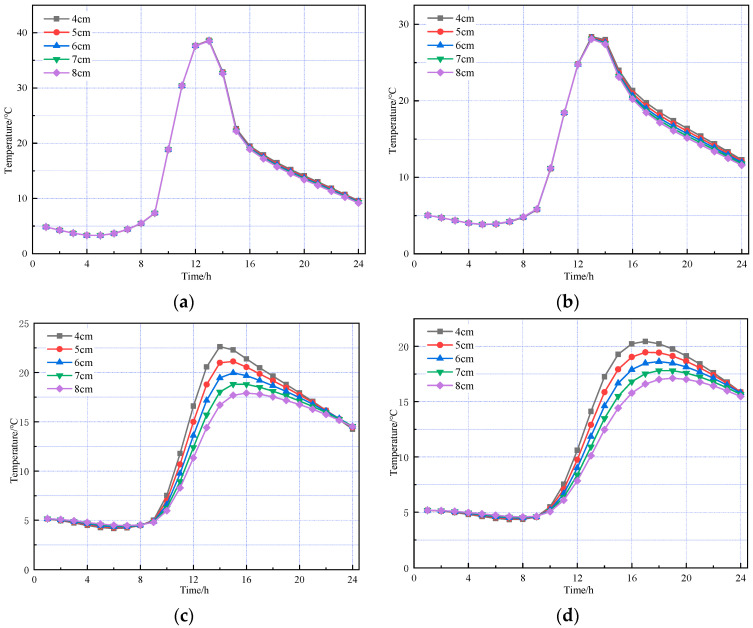
Temperature changes in pavement structures with different AC-20 thicknesses under winter temperatures. (**a**) The top of the SMA-13 layer. (**b**) The top of the AC-20 layer. (**c**) The top of the cement concrete layer. (**d**) The bottom of the cement concrete layer.

The thickness of the SMA-13 layer was kept constant, while the thickness of the AC-20 layer was set to different values. The thickness of the SMA-13 layer was kept constant at 4 cm, while the thickness of the AC-20 layer was set to 4, 5, 6, 7, and 8 cm, respectively. The results are shown in Figure 8 and Figure 9.

The above figures show that temperature variation trends for each pavement layer with varying AC-20 thicknesses are generally consistent in both summer and winter. The temperature field of the asphalt concrete layer is primarily influenced by environmental factors, such as solar radiation and atmospheric temperature. The temperature at the top surface of the SMA-13 layer remains essentially unaffected by AC-20 layer thickness, as pavement surface temperature is determined solely by external environmental conditions and the intrinsic properties of the asphalt concrete material. The thickness of the AC-20 layer has a minimal impact on the temperature of its top surface, showing only slight variations. In contrast, the cement concrete layer is more significantly affected by changes in AC-20 thickness; as the AC-20 thickness increases, the cement concrete layer temperature decreases accordingly.

**Figure 10:**
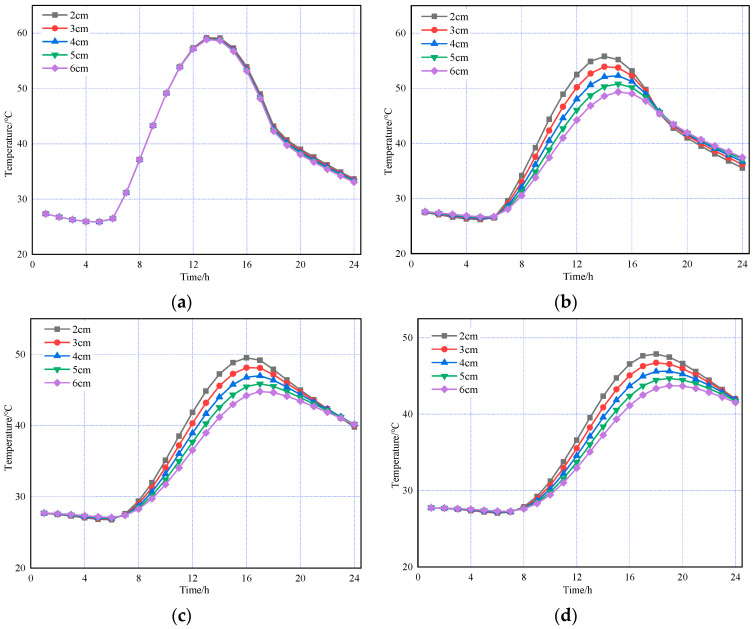
Based on the temperature changes in pavement structures with different SMA-13 thicknesses under summer temperatures. (**a**) The top of the SMA-13 layer. (**b**) The top of the AC-20 layer. (**c**) The top of the cement concrete layer. (**d**) The bottom of the cement concrete layer.

Next, the AC-20 layer thickness was fixed at 6 cm while the SMA-13 layer thickness varied at 2, 3, 4, 5, and 6 cm. The results are presented in Figure 10.

The above figures indicate that, in both summer and winter, changes in the asphalt concrete layer thickness have a minimal impact on the temperature field. The temperature of the asphalt concrete layer is mainly influenced by the atmospheric environment. In contrast, the temperature of the cement concrete layer decreases as the asphalt concrete layer thickness increases. Thus, the cement concrete layer’s temperature field is affected not only by the atmospheric environment but also by the asphalt concrete layer thickness.

## 4. Mechanical Response Analysis Under Moving Loads

This chapter explores the mechanical response of pavement structures under moving loads. This study employs a parametric analysis to comprehensively evaluate variables including vehicle load, slope gradients, braking coefficient and structural layer thickness. This study comparatively analyzes the stress–strain distribution characteristics within the structure under different working conditions and reveals how various factors influence the stress–strain distribution of the structure.

### 4.1. Axle Load

Currently, China’s designed standard axle load is 100 kN. Nevertheless, traffic volume survey data indicate that vehicles with an overload rate ranging from 100% to 200% constitute over 50% of the total. Vehicle overloading is a major cause of pavement damage. When using deflection value and tensile stress at the bottom of the asphalt layer as indicators, a single pass by a vehicle with an axle load of 300 kN is equivalent to 119 passes by a standard axle load vehicle. When using tensile stress in the semi-rigid material layer as the design indicator, a single pass by a vehicle with an axle load of 300 kN is equivalent to 6561 passes by a standard axle load vehicle [36].

**Figure 11:**
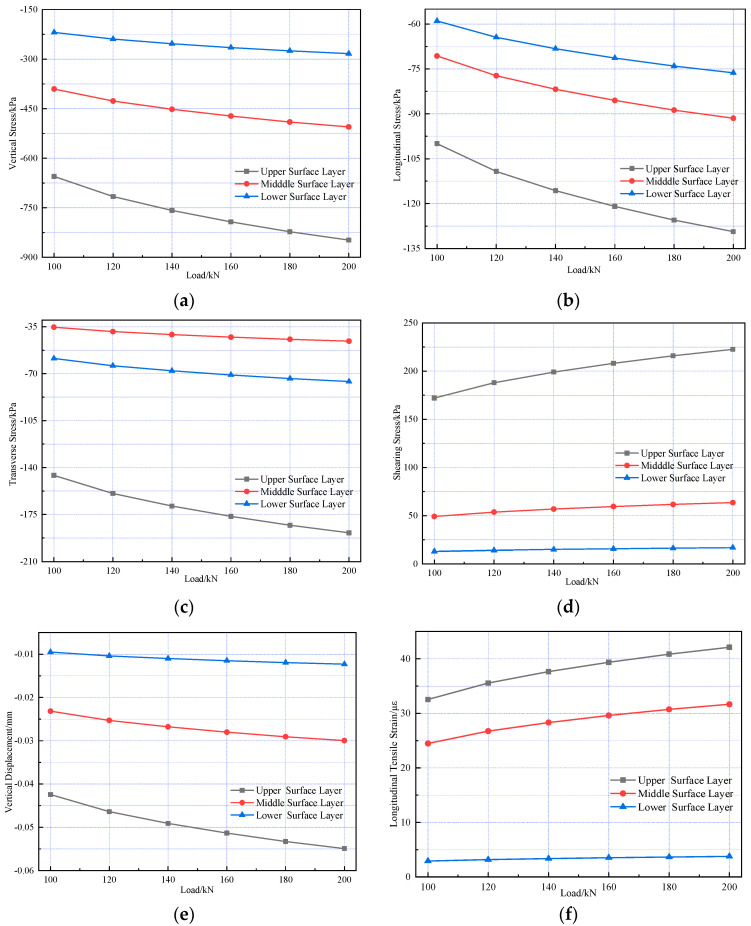
Variations in pavement mechanical response under different axle loads. (**a**) The vertical stress variation. (**b**) The longitudinal stress variation. (**c**) The transverse stress variation. (**d**) The shearing stress variation. (**e**) The vertical displacement variation. (**f**) The longitudinal strain variation.

Mechanical response analyses were conducted under standard axle load and 20%, 40%, 60%, 80%, and 100% overload conditions, respectively. Figure 11 illustrates the internal stress and strain within the pavement structure.

Figure 11 shows that as vehicle axle load increases, the peak values of vertical, longitudinal, and transverse stresses at the bottom of each structural layer in the composite pavement structure, along with longitudinal strain and surface vertical displacement responses, exhibit linear growth with load magnitude. Additionally, the amplitude of peak values for each index increasing with load is approximately 29%, showing basic consistency across all indices.

### 4.2. Slope Gradient

**Figure 12:**
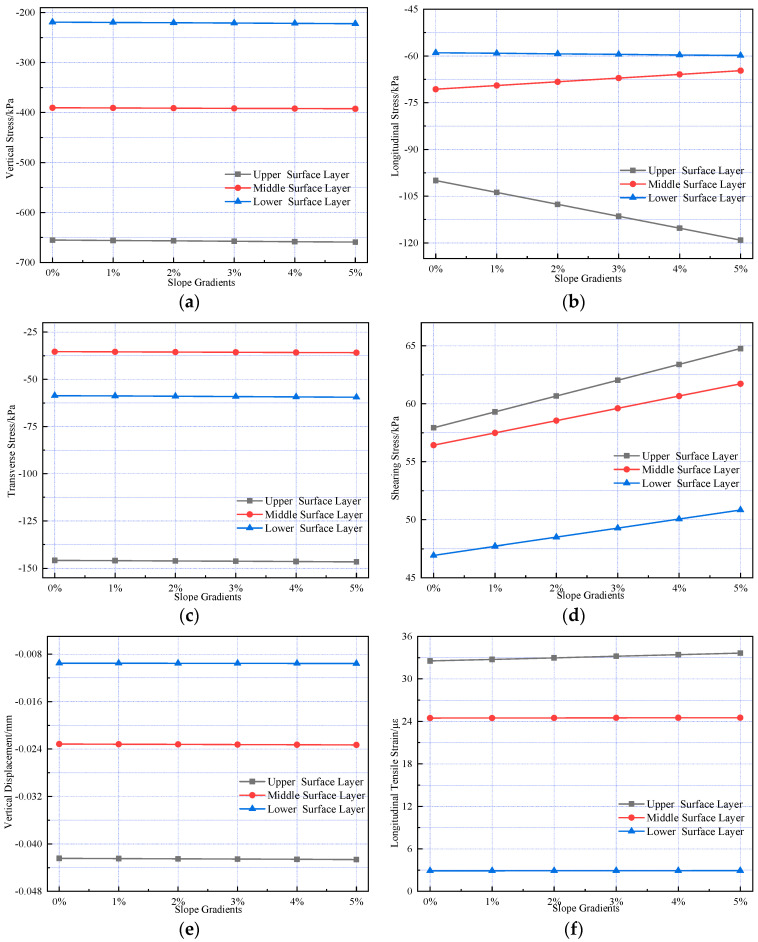
Variations in pavement mechanical response under different slope gradients. (**a**) The vertical stress variation. (**b**) The longitudinal stress variation. (**c**) The transverse stress variation. (**d**) The shearing stress variation. (**e**) The vertical displacement variation. (**f**) The longitudinal strain variation.

To investigate the effect of varying slope gradients on the internal mechanical responses of pavement structures in long longitudinal slope sections, slope gradients of 1%, 2%, 3%, 4%, and 5% were selected for analysis. Calculations were performed for interlayer vertical tensile stress, longitudinal tensile stress, transverse tensile stress, longitudinal shear stress, transverse shear stress, vertical displacement, and longitudinal tensile strain; the results are presented in Figure 12.

Figure 12 indicates that slope gradients have negligible effects on vertical stress, transverse tensile stress, shearing stress, vertical displacement, and longitudinal tensile strain in the composite pavement structure. As the slope gradient increases, the longitudinal tensile stress at the bottom of the cement concrete layer increases gradually. The longitudinal tensile stress at the bottom of the SMA-13 layer exhibits the largest increase (19.14%), whereas the longitudinal tensile stress at the bottom of the AC-20 layer decreases as the slope gradient increases. Longitudinal shearing stress in each structural layer increases with slope gradient, and the amplitude of increase decreases gradually with depth, ranging from 11.80% to 8.31%. As the slope gradient increases, the rise in horizontal load induces higher interlayer shearing stress within the pavement structure, rendering the road susceptible to distresses such as rutting, shoving, and bumping.

### 4.3. Braking Coefficient

**Figure 13:**
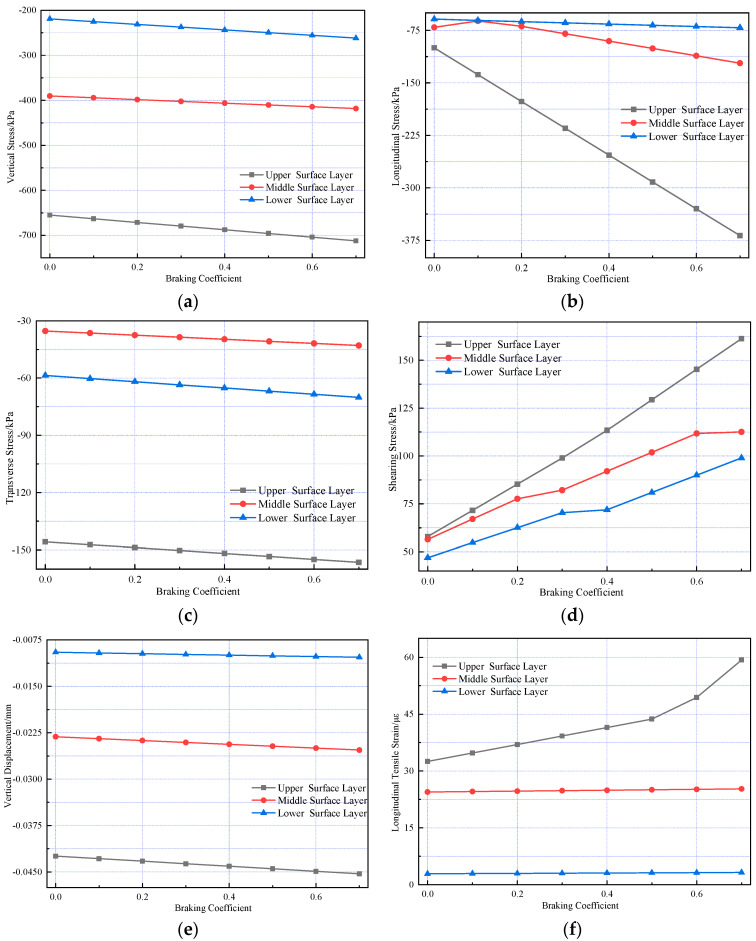
Variations in pavement mechanical response under different braking coefficients. (**a**) The vertical stress variation. (**b**) The longitudinal stress variation. (**c**) The transverse stress variation. (**d**) The shearing stress variation. (**e**) The vertical displacement variation. (**f**) The longitudinal strain variation.

During vehicle operation, tires impose not only vertical but also horizontal loads on the pavement due to frictional interaction with the road surface. Although such horizontal forces are generally minimal and often neglected, they can become significant under specific conditions such as long longitudinal slopes and intersections, where frequent braking intensifies the loading effect. To evaluate how varying braking intensities influence pavement behavior, braking coefficients ranging from 0 to 0.7 were examined, and the internal mechanical response of the pavement structure was computed accordingly. The results are presented in Figure 13.

Figure 13 shows that the braking coefficient significantly affects the vertical compressive stress, transverse tensile stress, shearing stress, vertical displacement, and longitudinal tensile strain in the composite pavement structure. As the braking coefficient increases, shearing stress and longitudinal tensile strain exhibit significant changes. Higher braking coefficients tend to induce shoving and bulging, resulting in shear failure of the pavement.

### 4.4. Thickness

**Figure 14:**
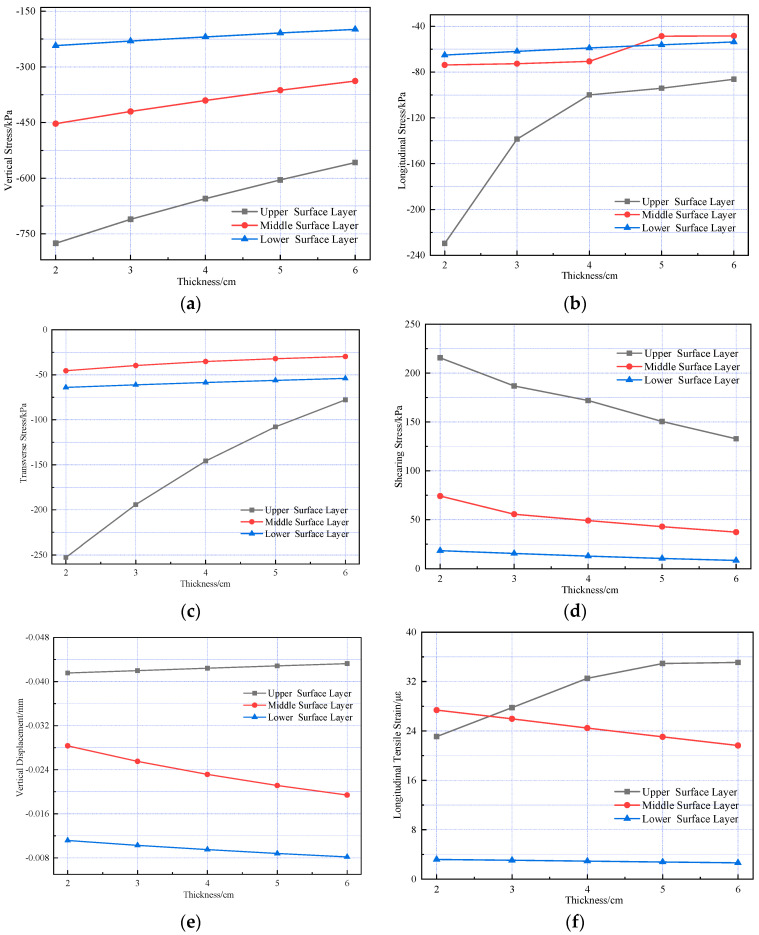
Changes in the internal mechanical response of pavement structures under different SMA-13 thickness conditions. (**a**) Vertical stress. (**b**) Longitudinal stress. (**c**) Transverse stress. (**d**) Shearing stress. (**e**) Vertical displacement. (**f**) Longitudinal strain.

**Figure 15:**
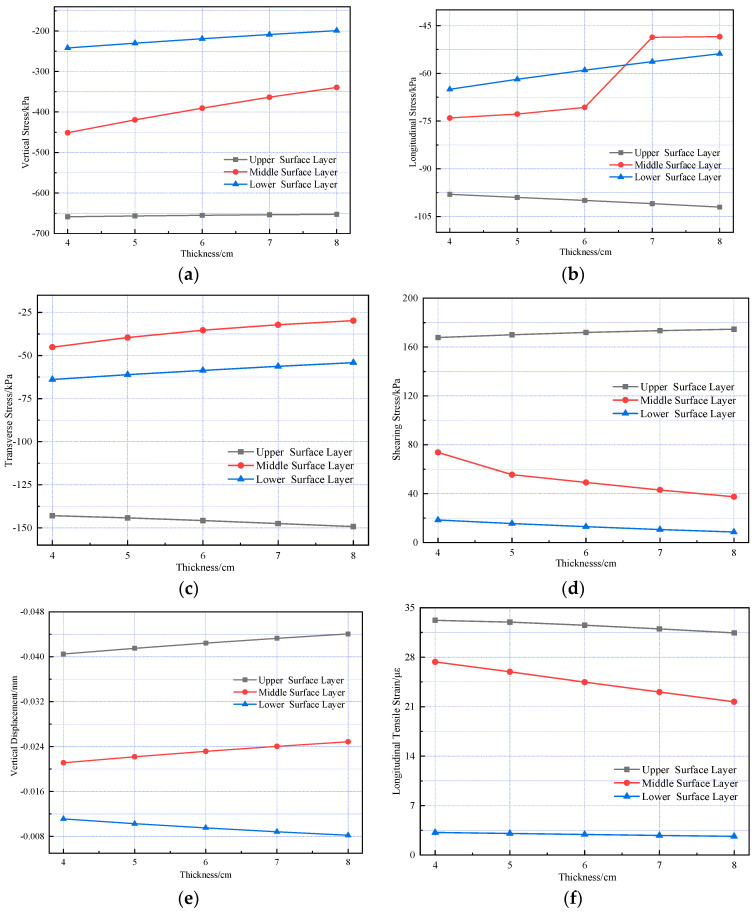
Changes in the internal mechanical response of pavement structures under different AC-20 thickness conditions. (**a**) Vertical stress. (**b**) Longitudinal stress. (**c**) Transverse stress. (**d**) Shearing stress. (**e**) Vertical displacement. (**f**) Longitudinal strain.

Additionally, to further investigate how asphalt layer thickness affects the internal mechanical response of the pavement structure, the mechanical response variation patterns of the pavement structure under different thicknesses of SMA-13 and AC-20 layers were analyzed separately. The same scheme used in the pavement structure temperature field was adopted: the SMA-13 layer thickness was fixed at 4 cm, with AC-20 layer thicknesses set as 4 cm, 5 cm, 6 cm, 7 cm, and 8 cm; the AC-20 layer thickness was fixed at 6 cm, with SMA-13 layer thicknesses set as 2 cm, 3 cm, 4 cm, 5 cm, and 6 cm. The results are presented in Figure 14 and Figure 15.

As shown in the figure, the thickness variation in the upper surface layer influences the pavement’s mechanical response more significantly than that of the middle surface layer. During pavement structure design, the thickness of the upper surface layer should be optimized based on site-specific conditions to minimize stress and strain within the structure and extend the service life of the pavement surface layer.

## 5. Conclusions

For asphalt pavements on long longitudinal slopes, existing studies often overlook the effects of temperature fields and fail to elucidate how various factors influence pavement mechanical responses. In this study, a composite pavement structure model incorporating temperature field effects was established in ABAQUS using air temperature data from Nanning, China. A moving load that mimics real vehicle characteristics was applied. Based on this model, parametric analyses were conducted with respect to axle load, braking coefficient, slope gradient, and structural layer thickness to characterize the thermo-mechanical response of asphalt pavements on long downhill sections. The main findings are as follows:

(1) The vertical, longitudinal, and transverse stresses within the pavement structure gradually decrease with increasing depth, with more significant attenuation observed in the middle and lower layers. Taking vertical stress under a standard axle load as an example, the bottom of the surface layer exhibits a stress value of 655.13 kPa, representing only a 6.5% reduction, while the middle and lower layers exhibit stresses of 390.5 kPa and 219.1 kPa, respectively, both reduced by over 40%.
(2) As the axle load increases from 100 kN to 200 kN, the peak responses of vertical stress, longitudinal stress, transverse stress, longitudinal strain, and vertical displacement at the bottom of each layer exhibit a linear relationship with the load magnitude. The increases in these peak values are generally consistent across indicators, with an average increase of approximately 29%.
(3) The shear stress within the pavement structure is more sensitive to slope changes. Compared with a flat road (0% slope), when the slope increases to 5%, the shear stresses at the bottom of the surface, middle, and lower layers increase by 12%, 9%, and 8%, respectively.
(4) The braking coefficient has a significant influence on the longitudinal stress, longitudinal strain, and shear stress at the bottom of the surface layer in composite pavement structures. Compared to the no-braking scenario, when the braking coefficient reaches 0.7, the increases in longitudinal stress, longitudinal strain, and shear stress at the bottom of the surface layer are 268%, 96%, and 178%, respectively.

This study reveals the thermo-mechanical response patterns of asphalt pavements on long longitudinal slopes, and clearly identifies the significant influence of axle load, slope gradient, braking coefficient, and structural layer thickness. The findings hold notable engineering implications. Under heavy loads and strong braking conditions, the internal stresses and strains in the surface layer significantly increase, making fatigue and shear failure more likely. Therefore, for the design of such special sections, asphalt mixtures with high shear strength and fatigue resistance should be prioritized, and the surface layer thickness should be appropriately increased to alleviate stress concentrations. Additionally, due to the increased shear stress caused by slope and braking effects, the interlayer bonding design should be strengthened—for example, by using high-performance tack coat materials or skid-resistant surfaces to enhance structural integrity.

Although this study established a three-dimensional finite element model incorporating temperature field effects and analyzed the impact of multiple key factors on the response of asphalt pavements on long slopes, certain limitations remain. First, the study primarily relies on numerical simulations to investigate thermo-mechanical behavior under different climatic conditions, lacking field tests to further support the findings. Moreover, the temperature field model is based solely on meteorological data from Nanning, limiting its applicability to other regions. To address these limitations, future work will focus on conducting field tests and extending validation to multiple environmental regions, in order to improve the accuracy of the simulation results and adjust model parameters based on actual operating conditions, thereby enhancing the model’s generalizability and practical applicability.

## Figures and Tables

**Table 1:** Mechanical properties of materials.

Material	Temperature (°C)	Elastic Modulus (MPa)	Poisson’s Ratio	Coefficient of Linear Thermal Expansion (10^−5^ K^−1^)
SMA-13	0	3180	0.25	3.29
	10	2830	0.25	3.61
	20	2470	0.25	3.93
	30	1820	0.30	4.25
	40	1554	0.35	4.57
	50	1130	0.40	4.89
	60	826	0.45	5.21
AC-20	0	3050	0.25	2.89
	10	2700	0.25	3.2
	20	2110	0.25	3.51
	30	1752	0.30	3.82
	40	1600	0.35	4.13
	50	940	0.40	4.44
	60	780	0.45	4.75
C30 Cement Concrete	—	33,000	0.15	1.00

**Table 2:** Thermal properties of materials.

Parameter	Asphalt Concrete	Cement Concrete
Thermal Conductivity (W/(m·K))	3.6	1.56
Density (kg/m^3^)	2300	2200
Specific Heat Capacity (J/(kg·K))	924.9	911.7
Solar Radiation Rate	0.90	
Pavement Emissivity	0.81	
Absolute Zero (°C)	−273	
Stefan–Boltzmann Constant (W/m^2^·k^4^)	5.67 × 10^−8^	

**Table 3:** Twenty-four-hour temperature profile for a typical summer day.

Time (h)	Temperature (°C)	Time (h)	Temperature (°C)	Time (h)	Temperature (°C)	Time (h)	Temperature (°C)
0	29.48	7	29.77	14	36.17	21	31.06
1	28.83	8	30.19	15	35.93	22	30.08
2	28.8	9	30.73	16	35.38	23	29.66
3	28.81	10	33.32	17	34.29	24	29.59
4	28.4	11	34	18	31.71		
5	28.36	12	34.44	19	31.45		
6	29.41	13	36.09	20	31.16		

**Table 4:** Twenty-four-hour temperature profile for a typical winter day.

Time (h)	Temperature (°C)	Time (h)	Temperature (°C)	Time (h)	Temperature (°C)	Time (h)	Temperature (°C)
0	8.85	7	6.02	14	12.67	21	9.8
1	7.73	8	6.33	15	12.9	22	9.04
2	7.6	9	6.48	16	13.03	23	8.74
3	7.36	10	9.12	17	12.95	24	8.67
4	6.45	11	9.7	18	10.56		
5	6.36	12	10.21	19	10.1		
6	6.07	13	12.36	20	9.86		

## Data Availability

The original contributions presented in this study are included in the article. Further inquiries can be directed to the corresponding author.
